# A Manual of the Modern Theory and Technique of Surgical Asepsis

**Published:** 1896-03

**Authors:** 


**A Manual of the Modern Theory and Technique of Surgical Asepsis.** By Carl Beck, M.D. Pp. 306. Philadelphia: W. B. Saunders. 1895.

In the frequent washing of patients before operation of the Hippocratic era, and the ablutions ordained by Mosaic rite, Dr. Beck sees early gleams of the modern aseptic treatment. It is a regrettable fact that many surgeons have not yet grasped the essential difference between antisepsis and asepsis, thinking that their responsibility ends when they have dipped their fingers in some potent chemical fluid, and to such this book may be commended; for in clear and intelligible language the doctrine of asepsis, the offspring of antisepsis, is set forth. The book is most essentially practical, and thus serves a very useful purpose; but on the theoretical side it will not be found to possess any further advantages to those who have already become familiar with other recent works on this important subject. Most of the remarks on anæsthesia and the surgical treatment of tuberculosis seem to us outside the domain of such a treatise, and might be omitted with advantage. The book is excellently illustrated, and orthographical errors are few; "natrum," however, occurs on p. 106. The type is good, the index most complete, but the binding is somewhat deficient.

